# ﻿Taxonomic revision of *Camellialangbianensis* (Theaceae) with four new synonyms

**DOI:** 10.3897/phytokeys.234.110218

**Published:** 2023-10-26

**Authors:** Dongwei Zhao

**Affiliations:** 1 Department of Forestry, College of Forestry, Central South University of Forestry and Technology, Changsha, 410004, China Central South University of Forestry and Technology Changsha China

**Keywords:** *Dankia*, endemic, golden camellia, Indochina

## Abstract

Based on analysis of morphologically diagnostic characters, *Camellialangbianensis*, a yellow camellia native to southern Vietnam, is taxonomically revised to include four new heterotypic synonyms: *C.decora*, *C.dongnaiensis*, *C.oconoriana* and *C.tadungensis*. *Camelliavidalii* is retained in the synonymy of *C.langbianensis*. Updated description and distribution map for *C.langbianensis* are provided.

## ﻿Introduction

Gagnepain (1939: 198) established *Dankia* Gagnep. for *Dankialangbianensis* Gagnep. The species was transferred into *Camellia* L. (Theaceae) by Hô (1991: 537) as *Camellialangbianensis* (Gagnep.) P.H. Hô. Accordingly, the monotypic genus *Dankia* became a heterotypic synonym of *Camellia* (Zhao et al. 2017a; Zhao 2022a), which had been overlooked in Ming (2000). Nevertheless, *Dankia* remains listed as an accepted genus in the family Theaceae on the Angiosperm Phylogeny Website (http://www.mobot.org/MOBOT/research/APweb/genera/theaceaegen.html). Sealy suggested that a columella was absent in the fruit of *D.langbianensis*, so *Dankia* was distinct from *Camellia*; however, subsequent investigations revealed that the capsule of *D.langbianensis* does bear a columella (Zhao 2019; Quach et al. 2021). Morphological investigations have not revealed any substantial differences between *Camellia* and *Dankia*. Both genera bear a generally globose capsule with the wingless seeds attached to the columella (Zhao 2019; Quach et al. 2021). Molecular phylogenetic analysis suggested that *C.vidalii*, a heterotypic synonym of *C.langbianensis* (Zhao 2019), was nested in the clade Piquetia within the monophyletic genus *Camellia* (Zhao et al. 2023). Therefore, it can be reasonable to conclude that *Dankia* is a synonym of *Camellia*, based on both morphological and molecular data.

Though more than 100 species of *Camellia* have been described since Ming’s (2000) latest monograph of the genus and most of them were derived from Vietnam (Zhao et al. 2023), recent research suggested that many of them were merely repeated names for the previously-published species (e.g. Zhao et al. [2017b]; Zhao [2019, 2021, 2022b]). Plants of *Camellia* can vary widely within a single species, a remarkable example is that hundreds of cultivars of *C.japonica* L., *C.sasanqua* Thunb. and *C.sinensis* (L.) Kuntze have been recorded (Wang et al. 2019). It would be unreasonable to suppose that a species should vary narrowly in nature, but broadly in cultivation. However, Quach et al. (2021) argued that *C.vidalii* could be distinguished from *C.langbianensis* by the indumenta of the flower parts, which suggested that *C.langbianensis* and *C.vidalii* were distinct species. Additionally, during a revision of camellias in Vietnam, I found that four previously described species (Orel 2006; Orel et al. 2013; Orel and Curry 2015) had a close relationship with *C.langbianensis*. They are discussed below in detail with a taxonomic revision of *C.langbianensis* provided.

## ﻿Material and methods

Taxonomic literature (e.g. Sealy [1958]; Chang [1981]; Hô [1991]; Ming [2000]), especially protologues of taxa (e.g. Gagnepain [1939]; Rosmann [1999]; Orel [2006]; Orel et al. [2013]; Orel and Curry [2015]), were studied. Types and additional specimens and/or their images conserved at Herbaria DLU, HN, HNU, K, KUN, L, NSW, P, PE, PHH, SGN, TCD, VFM, VNF, VNM and VNMN (acronyms based on Thiers 2023, continuously updated) were examined. Article 11.4 of the Shenzhen Code (Turland et al. 2018) was applied to evaluate the priority of the name of a species. Morphological characters were described or measured, based on collections and/or their images examined and the protologues to make comparisons. Geographic coordinate data were retrieved from the records on specimens and mapped using ArcMap 10.7 and then optimised in Adobe Illustrator.

## ﻿Taxonomic treatment

### ﻿ Camellia langbianensis

Taxon classificationPlantae

(Gagnep.) P.H. Hô, Cayco Vietnam 1(1): 537. 1991.

E2643EC9-1F14-5FE6-96BF-F1A66D86B9C5

≡ Dankialangbianensis Gagnep., Fl. Indo-Chine [P.H. Lecomte et al.] Suppl.: 198. 1939. Lectotype (designated by Zhao et al. [2017a: 173]): Vietnam. [Lam Dong]: entre B. dlé et Dankia, Langbiang, 1200–1300 m elev., 26 October 1930, *E. Poilane 18648* (P00754831! Image: https://science.mnhn.fr/institution/mnhn/collection/p/item/p00754831).
= Camelliavidalii Rosmann, Adansonia 21(2): 319. 1999. Holotype: Vietnam. Lam Dong: Bao Loc, 875 m elev., December 1998, *J.C. Rosmann et al. 981* (P00834283! Image: https://science.mnhn.fr/institution/mnhn/collection/p/item/p00834283).
= Camelliadongnaiensis Orel, Novon 16(2): 244. 2006. Syn. nov. Holotype: Vietnam. Lam Dong: unnamed tributary, the headwaters of Dong Nai River, 17 January 2004, *G. Orel et al. 21148* (NSW868472, image!).
= Camelliaoconoriana Orel, Curry & Luu, Edinburgh J. Bot. 70(3): 440. 2013. Syn. nov. Holotype: Vietnam. Lam Dong: unnamed mountain about 120 km SW of Dalat, 22 November 2010, *G. Orel & A.S. Curry 0720* (NSW900415, image!).
= Camelliadecora Orel, Curry & Luu, Pursuit Hidden Camellias Vietnam China 173. 2015. Syn. nov. Holotype: Vietnam. Ninh Thuan, 23 March 2009, *H.T. Luu et al. VNM 12381* (NSW901588, image!).
= Camelliatadungensis Orel, Curry & Luu, Pursuit Hidden Camellias Vietnam China 256. 2015. Syn. nov. Holotype: Vietnam. Dak Nong: Ta Dung Nature Reserve, 11 January 2011, *H.T. Luu et al. TD 264* (NSW901888, image!).

#### Description.

Shrubs 4–6 m tall. ***New branchlets*** puberulous; ***terminal buds*** pubescent. ***Petioles*** 6–17 mm long, puberulous to glabrous; ***leaf blades*** narrowly elliptic, oblong or ligulate, 15–40 × 2.5–15 cm, coriaceous, abaxially yellowish or pale green and puberulous, adaxially dark green and glabrous, mid-rib raised on both surfaces, secondary veins 14–27 on each side of mid-rib, abaxially raised and adaxially impressed, base cuneate, obtuse, rounded or subcordate, margin nearly entire or sparsely serrulate, apex attenuate. ***Flowers*** solitary or paired, borne in the axils of leaves or on short bracteate shoots; short shoots bearing 3–5 bracts subtending flowers; bracts caducous; flowers 2.5–4.5 cm in diam. ***Pedicels*** 2–5.5 cm long, puberulous to glabrous. ***Bracteoles*** 2–4, alternate, narrowly ovate or deltate, 1.5–6 × 1.5–3 mm, outside puberulous, inside glabrous or puberulous, margin ciliolate. ***Sepals*** 5–6, broadly ovate or ovate, 4–7 × 4–12 mm, outside puberulous to pubescent, inside glabrous or puberulous, margin ciliolate. ***Petals*** 7–9 in 2 whorls, yellow or with pale red fringe, broadly obovate or elliptic, 1–2.5 × 1–2.2 cm, outer surface pubescent to puberulous, inner surface glabrous or puberulous. ***Stamens*** 1–2 cm long, outer filaments basally connate for 3–4 mm, adnate to petals for 1–2 mm, glabrous or basally pubescent. ***Ovary*** oblate or ovoid, densely pubescent. ***Styles*** 3–6, distinct, 1–2.5 cm long, pubescent or gradually becoming glabrous apically. ***Capsule*** oblate, 5–7 cm in diam., 2–3.5 cm in height; pericarp 0.5–3 mm thick. ***Seeds*** reddish-brown or black, hemispherical or polyhedral, 1.5–2 cm in diam., glabrous and shiny.

#### Phenology.

Flowering November–April, fruiting April–October.

#### Distribution.

**Figure 1.**
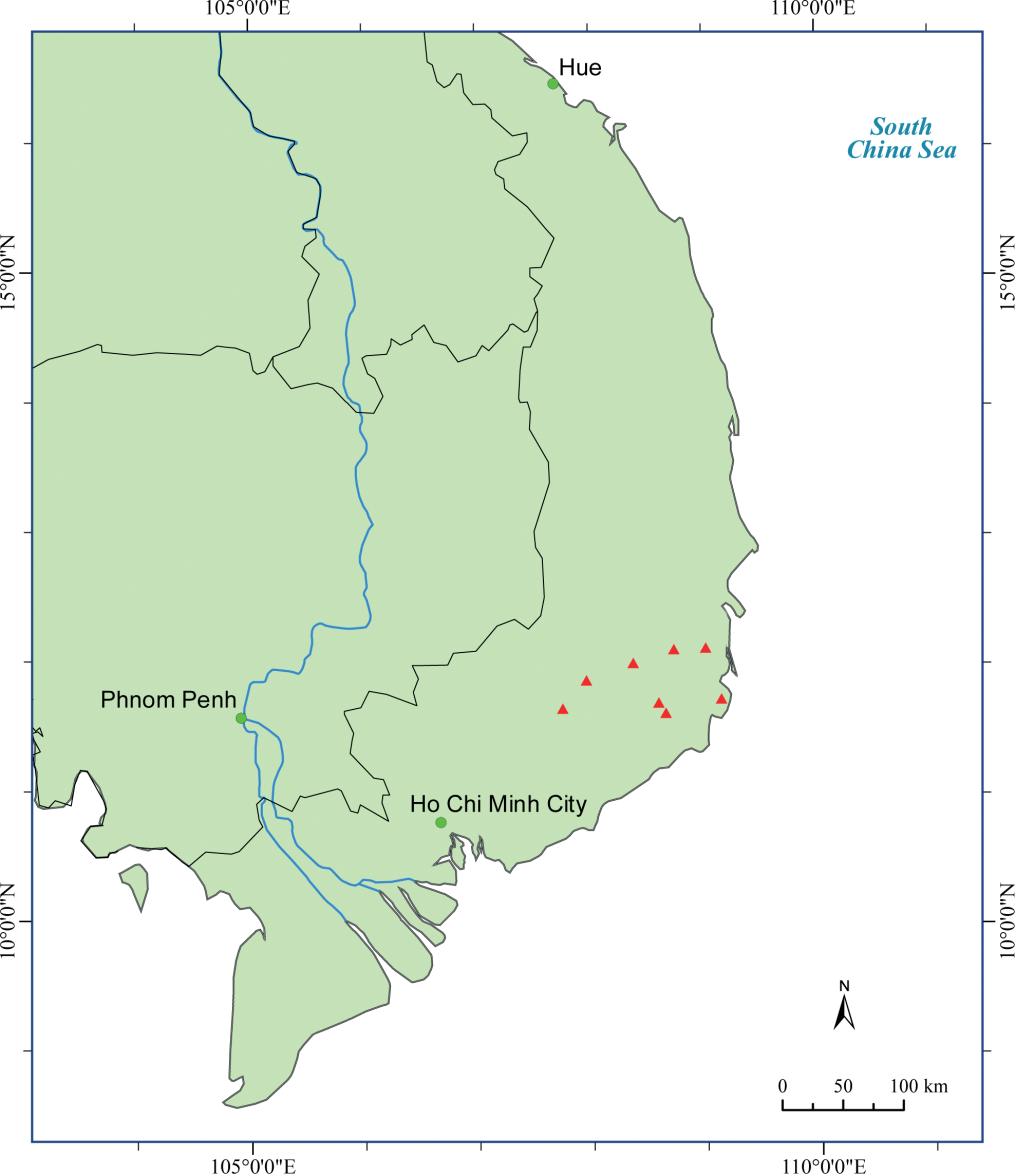
Geographic distribution of *Camellialangbianensis* (red triangle) in Vietnam.

Endemic to southern Vietnam, in Dak Nong, Khanh Hoa, Lam Dong and Ninh Thuan Provinces (Fig. 1).

#### Habitat.

Evergreen forest, 750–1800 m elev.

#### Additional specimens examined.

Vietnam. Khanh Hoa: Cam Lam, Hon Ba, 8 April 2012, *L.H. Truong & T. Gioi KH 86, KH 87 & KH 88* (SGN), 8 April 2013, *L.H. Truong & T. Gioi KH 1140* (SGN). Lam Dong: Bao Loc, Pu Sapoum près Mt. ageicole de Blao, 1000–1100 m elev., 10 January 1934, *E. Poilane 23790* (P04500357, image: https://science.mnhn.fr/institution/mnhn/collection/p/item/p04500357); Dam Ri, 11 January 2012, *DL 12.01.02* (DLU), 1 December 2012, *DL 12.12.02* (DLU), 31 January 2015, *DL 15.01.03* (DLU), 11°38'29"N, 107°44'25"E, 780–830 m elev., 30 November 2015, *D.W. Zhao & L.V. Dung 124* (TCD), *125* (KUN, PHH, TCD) & *126* (TCD); Don Duong, Pro, 15 February 2014, *DL 14.02.01*, *DL 14.02.02*, *DL 14.02.03* (DLU), *DL 15.10.08* (DLU); massif du Braïan près de Djiling, 1700–1800 m elev., 17 January 1935, *E. Poilane 23959* (P05312544, image: https://science.mnhn.fr/institution/mnhn/collection/p/item/p05312544), 1200–1400 m elev., 3 February 1935, *E. Poilane 24105* (P06838121, image: https://science.mnhn.fr/institution/mnhn/collection/p/item/p06838121). Ninh Thuan: Ninh Hai, Nui Chua, 22 March 2009, *Truong & Dat NC 198* (SGN), March 2010, *Luu 749* (SGN), March 2011, *Luu 750* (SGN), 20 April 2012, *Luu 736* (SGN); Ninh Son, Phuoc Binh, 37 km to NE from Dalat City, 12°6'N, 108°43'E, 1300–1400 m elev., 2 April 1997, *L. Averyanov et al. VH 3561* (HN, P05191415, image: https://science.mnhn.fr/institution/mnhn/collection/p/item/p05191415; P05247468, image: https://science.mnhn.fr/institution/mnhn/collection/p/item/p05247468).

#### Notes.

The diagnostic morphological differences amongst the type materials of *C.decora*, *C.dongnaiensis*, *C.langbianensis*, *C.oconoriana*, *C.tadungensis* and *C.vidalii* are shown in Table 1. Quach et al. (2021) argued that *C.vidalii* could be distinguished from *C.langbianensis* by its glabrous pedicel, basal part of filaments, upper part of styles and inside surfaces of the bracteoles, sepals and petals, whereas the latter was hairy on the parts listed. However, the original materials of *C.langbianensis* bear a glabrous to sparsely puberulous pedicel and its bracteoles are glabrous or sparsely puberulous on the inside surface (isolectotypes: K000704329; P00754832, image: https://science.mnhn.fr/institution/mnhn/collection/p/item/p00754832). The specimens *L. Averyanov et al. VH 3561* at HN and P, cited as *C.langbianensis* in Quach et al. (2021), bear nearly glabrous sepals and much less hairy petals. By contrast, the filaments of the holotype of *C.vidalii*, *J.C. Rosmann 981* (P00834283), are basally pubescent. The persistent sepals of *L.H. Truong & T. Gioi KH 1140* at SGN, a specimen that is conspecific with *C.vidalii*, are hairy on the inside surface.

**Table 1.** Morphological comparisons of the types of *Camelliadecora*, *C.dongnaiensis*, *C.langbianensis*, *C.oconoriana*, *C.tadungensis* and *C.vidalii*.

Character	*C.decora*	*C.dongnaiensis*	*C.langbianensis*	*C.oconoriana*	*C.tadungensis*	*C.vidalii*
Length of petiole	10–15 mm	10–15 mm	10–17 mm	10–15 mm	10–14 mm	9–13 mm
Size of leaf blade	25–30 × 8–11 cm	29–46 × 9–15 cm	21–28 × 3–5.5 cm	32–35 × 5.5–8.5 cm	24–34 × 6–9 cm	20–36 × 4–7 cm
Pairs of secondary veins	16–19	15–27	14–16	19–24	14–16	17–22
Indumentum of pedicel	glabrous	glabrous to puberulous	glabrous to sparsely puberulous	glabrous	glabrous	glabrous
Indumentum of sepals	outside pubescent, inside glabrous	outside pubescent, inside glabrous	outside pubescent, inside puberulous	outside puberulous, inside glabrous	outside pubescent, inside glabrous	outside puberulous, inside glabrous
Indumentum of petals	outside pubescent, inside glabrous	outside pubescent, inside glabrous	outside pubescent, inside puberulous	outside pubescent, inside glabrous	outside pubescent, inside glabrous	outside pubescent, inside glabrous
Indumentum of filaments	glabrous	Unknown	Unknown	glabrous	glabrous	basally puberulous
Number of styles	3–4	5–6	5	3–5	5–6	4–5
Indumentum of styles	pubescent	glabrous	basally pubescent	basally pubescent	basally pubescent	pubescent
Phenology	flowering March	flowering January	flower buds in October	flowering November	flowering January	flowering December

When more collections are examined, it is hard to ignore the morphological variations of the plants represented by *C.langbianensis* and its synonyms listed above (Table 1). The size of leaves and indumenta of pedicel, sepals, petals and filaments usually vary continuously amongst individuals and a clearly diagnostic breaking point is generally absent. For example, the size of the leaves increases from the type of *C.langbianensis* to that of *C.dongnaiensis*, with those of *C.decora*, *C.oconoriana*, *C.tadungensis* and *C.vidalii* locating between them and overlapping with each other. The type of *C.langbianensis* might be much hairier on the sepals and petals than those of the synonyms recognised above (Table 1; Quach et al. [2021]). The morphological variation is, however, hardly convincing to differentiate species in *Camellia* because this kind of variation can also be found within other taxa, such as C.sinensisvar.pubilimba Hung T. Chang and *C.lanceolata* (Blume) Seem. (Ming 2000). Orel (2006) argued that *C.dongnaiensis* was unique by its yellow petals with pink margin; nevertheless, the fringed pink petals can also be found in *C.langbianensis* (Quach et al. 2021). Therefore, I retain my previous treatment (Zhao 2019) that *C.vidalii* is a heterotypic synonym of *C.langbianensis* before more data are available and treat *C.decora*, *C.dongnaiensis*, *C.oconoriana* and *C.tadungensis* as new heterotypic synonyms of *C.langbianensis*.

*Camellialangbianensis* is characterised by its generally large leaves, long pedicels, persistent bracteoles and sepals, yellow petals hairy outside, distinct styles, hairy ovaries and glabrous and shiny seeds (Table 1). As a member of sect. Piquetia (Pierre) Sealy, it is closely-related with *C.dalatensis* V.D. Luong, Ninh & Hakoda (Tran et al. 2012) and *C.piquetiana* (Pierre) Sealy (Zhao et al. 2023). *Camellialangbianensis* can be distinguished from *C.dalatensis* by its nearly glabrous branchlets, petiole and abaxial surface of the leaf blade, whereas the latter are pubescent on those parts mentioned. *Camelliapiquetiana* bears a shorter pedicel and red petals, while *C.langbianensis* has a longer pedicel and yellow petals.

## Supplementary Material

XML Treatment for Camellia langbianensis

## ﻿Additional information

### Author ORCIDs

Dongwei Zhao https://orcid.org/0000-0002-7761-7127

### Data availability

All of the data that support the findings of this study are available in the main text.
